# The crystal structure of the ammonium salt of 2-amino­malonic acid

**DOI:** 10.1107/S2053229624005576

**Published:** 2024-06-19

**Authors:** Dirk Hollenwäger, Alexander Nitzer, Valentin Bockmair, Andreas J. Kornath

**Affiliations:** a Department Chemie, Ludwig-Maximilians Universität, Butenandtstrasse 5-13 (Haus D), D-81377 München, Germany; University of Sydney, Australia

**Keywords:** crystal structure, 2-amino­malonic acid, Raman, NMR, peptide synthesis

## Abstract

The salt ammonium 2-am­ino­mal­on­ate (systematic name: ammonium 2-aza­niumyl­propane­dioate), NH_4_^+^·C_3_H_4_NO_4_^−^, was synthesized in diethyl ether from the starting materials malonic acid, ammonia and bromine. The salt was recrystallized from water as colourless blocks. In the solid state, intra­molecular medium–strong N—H⋯O, weak C—H⋯O and weak C—H⋯N hydrogen bonds build a three-dimensional network.

## Abstract 2

2-Amino­malonic acid is particularly inter­esting for the synthesis of peptides and amino acids due to its two carboxyl groups. The crystal structure reported here is the first single-crystal X-ray diffraction measurement of this com­pound or one of its salts.

## Introduction

The first synthesis of 2-amino­malonic acid was in 1864 and described by Bayer (Beaujon & Hartung, 1953 ▸). In 1902, Ruhemann and Orton investigated the preparation with nitro­malonamide as a starting material and a reduction with amalgam (Beaujon & Hartung, 1953 ▸). In 1902, Lütz used halogenated malonic acid and ammonia as the starting materials to obtain 2-amino­malonic acid as the product (Beaujon & Hartung, 1953 ▸). To obtain a much purer product, Hartung invented in 1952 a distillation in a vacuum with a palladium–charcoal catalyst. 2-Amino­malonic acid was obtained in a yield of 80–90% (Beaujon & Hartung, 1953 ▸).
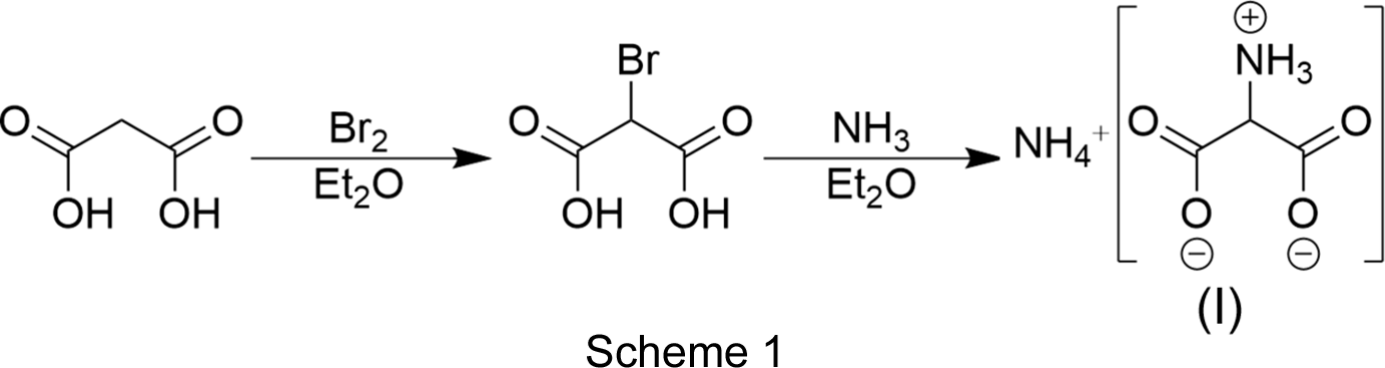


2-Amino­malonic acid is used as a complexone in medicine, environmental technology and chemistry due to it being a member of the amino polycarb­oxy­lic acid group of substances (Anderegg *et al.*, 2005 ▸). In 1945, G. Schwarzenbach introduced the name ‘complexones’ for laboratory-synthesized com­pounds which are close to amino acids (Anderegg *et al.*, 2005 ▸). Well-known representatives of complexones are, for example, EDTA (ethyl­enedi­amine­tetra­acetic acid), DTPA (di­ethyl­enetri­amine penta­acetate) or TETA (tri­ethyl­ene­tetra­mine) (Anderegg *et al.*, 2005 ▸). These com­pounds are built with a nitro­gen-containing moiety which enables their use as ligands.

The corresponding acids of 2-amino­malonic acid and its salts are of particular inter­est because of their two carboxyl groups, one of which can be deca­rboxylated to form a chiral centre (Zheng *et al.*, 2023 ▸). Like other complexones, 2-amino­malonic has a nitro­gen moiety and other functional groups that are very suitable for binding complexes (Anderegg *et al.*, 2005 ▸). The zwitterionic character is similar to that of amino acids and makes it possible to use it as a ligand at different pH values.

## Experimental

### Synthesis and crystallization

Malonic acid (10.4 g, 0.1 mmol) and diethyl ether (100 ml) were added to a dried Schlenk flask. The mixture was cooled to 273 K and bromine (16.0 g, 0.1 mol) was added under stirring over a period of 40 min. The mixture was warmed to room temperature and stirred for a further 40 min. Aqueous am­monia (100 ml, 25%) was added slowly under stirring. The solvent was removed in a vacuum. The product was obtained as a white-to-light-yellow solid product. The synthesis route is shown in Scheme 1.

### Analysis (X-ray, Raman and NMR)

We investigated and characterized salt (I) by single-crystal X-ray diffraction, Raman spectroscopy and NMR spectroscopy. Complete data and devices for the X-ray measurements are listed in the CIF in the supporting information. Low-tem­perature Raman spectroscopic studies were performed using a Bruker MultiRAM FT–Raman spectrometer with an Nd:YAG laser excitation (λ = 1064 cm^−1^) under vacuum at 77 K. For a measurement, the synthesized com­pound was transferred to a cooled glass cell. A Bruker AV400TR spectrometer was used for the ^1^H, ^13^C and ^14^N NMR measurements.

### Refinement

Crystal data, data collection, and structure refinement details are summarized in Table 1 ▸.

## Results and discussion

### Single-crystal X-ray diffraction

**Figure 1:**
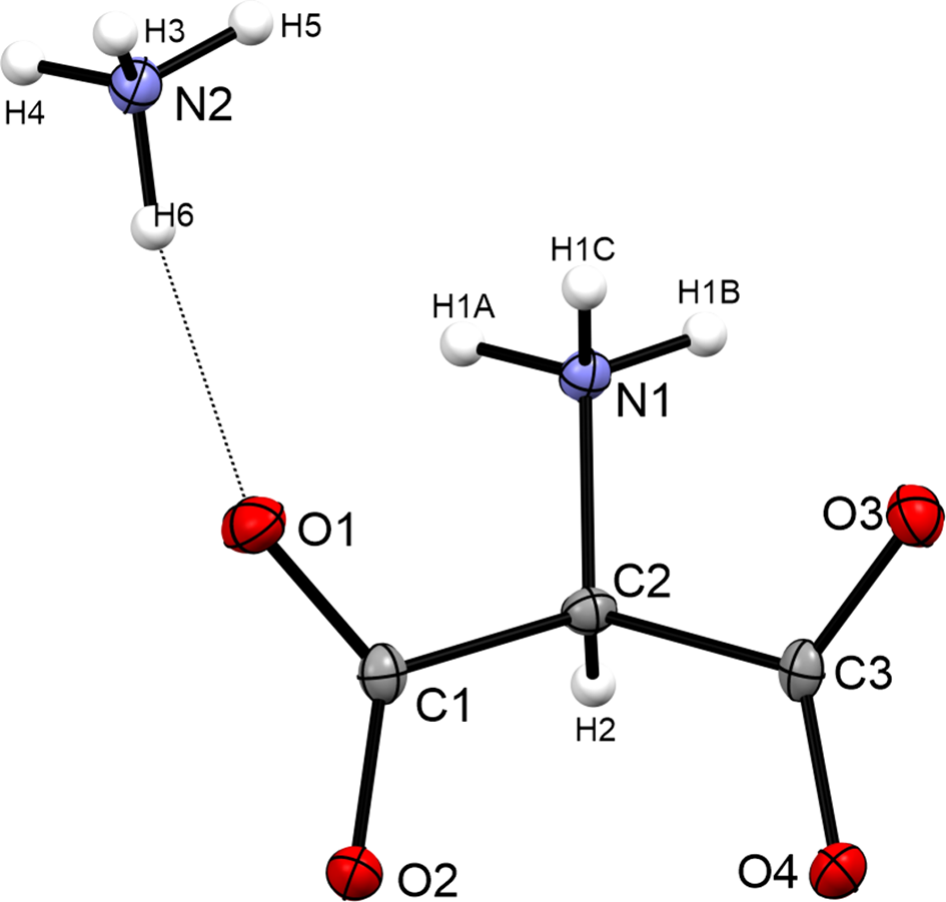
The asymmetric unit of salt (I), with displacement ellipsoids drawn at the 50% probability level.

Herein, we present the first single-crystal X-ray diffraction analysis of the salt ammonium 2-am­ino­mal­on­ate, NH_4_^+^·C_3_H_4_NO_4_^−^, (I), as a zwitterion. The salt crystallizes in the ortho­rhom­bic space group *Pbca* with eight formula units per unit cell. The asymmetric unit is shown in Fig. 1 ▸. The C—C bonds are 1.5394 (18) (C1—C2) and 1.5485 (18) Å (C2—C3). The C—C bonds are significantly elongated compared to the median of the average C*sp*^2^—C*sp*^3^ hybridized bond (1.475–1.522 Å) determined by X-ray diffraction (Allen *et al.*, 1987 ▸). The C2—N1 bond [1.4821 (16) Å] is in the same range as the median of an average C*sp*^3^—N*sp*^3^ hybridized bond (1.488 Å) and that of glycine (1.484 Å) (Allen *et al.*, 1987 ▸; Iitaka, 1960 ▸). The shorter C—O bond lengths of 1.2483 (16) (C1—O1) and 1.2462 (17) Å (C3—O3) are significantly elongated by approximately 0.015 Å compared to the shorter C—O bond in β-glycine (1.233 Å) (Iitaka, 1960 ▸). The longer C—O bonds are 1.2657 (16) (C1—O2) and 1.2597 (16) Å (C3—O4). In com­pari­son to β-glycine (1.257 Å), the C1—O2 bond is slightly elongated (Iitaka, 1960 ▸).

The carbon chain has a C1—C2—C3 angle of 113.00 (10)° and is only slightly magnified compared to the starting material [111.3 (1)°; Jagannathan *et al.*, 1994 ▸]. The O1—C1—O2 [124.87 (12)°] and O3—C3—O4 [127.55 (12)°] angles are only slightly influenced by the NH_3_ moiety compared to the starting material [O1—C1—O2 = 124.8 (1)° and O1—C1—O2 = 123.3 (2)°]. The N1—C2—C1 angle is 109.56 (10)° and the N1—C2—C3 angle is 109.98 (10)°. The torsion angles are −2.96 (16) (O1—C1—C2—N1), 175.49 (11) (O2—C1—C2—N1), 10.44 (15) (O3—C3—C2—N1) and −169.89 (10)° (O4—C3—C2—N1).

**Figure 2:**
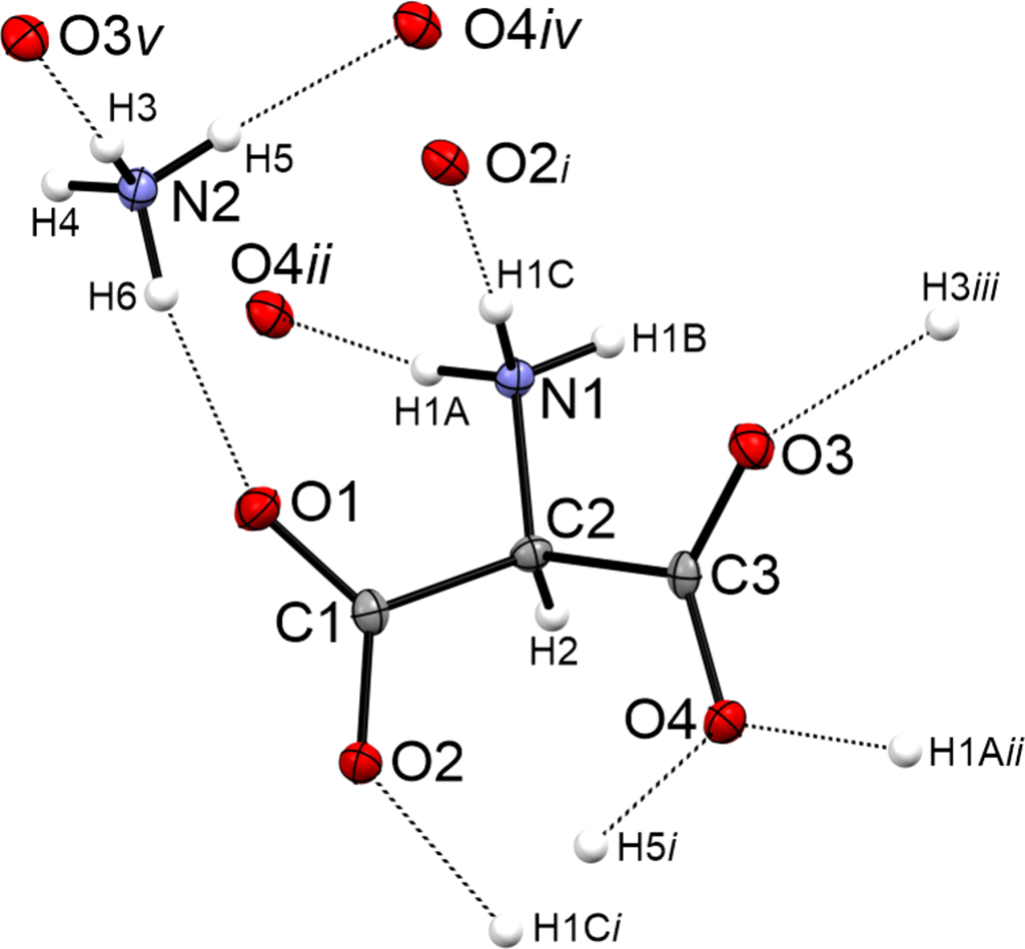
Hydrogen bonds in the crystal structure of salt (I), with displacement ellipsoids drawn at the 50% probability level. [Symmetry codes: (i) *x* + 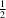, *y*, −*z* + 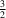; (ii) −*x* + 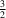, −*y* + 1, *z* − 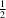; (iii) −*x* + 1, *y* + 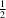, −*z* + 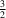; (iv) *x* − 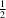, *y*, −*z* + 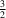; (v) −*x* + 1, *y* − 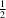, −*z* + 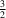.]

The crystal structure of salt (I) displays a three-dimensional network built of moderate N—H⋯O hydrogen bonds, according to the classification of Jeffrey (1997 ▸). Fig. 2 ▸ shows the hydrogen bonds in the crystal structure. The hydrogen bonds are listed in the CIF in the supporting information. The strongest hydrogen bond, N2—H6⋯O1, is in the asymmetric unit with an N⋯O distance of 2.803 (2) Å. The crystal struc­ture builds chains *via* N1—H1*C*⋯O2^i^ [2.928 (1) Å] and N2—H5⋯O4^iv^ [2.908 (2) Å] hydrogen bonds. The chains are connected *via* N2—H3⋯O3^v^ [2.832 (2) Å] and N1—H1*A*⋯O4^ii^ [2.822 (2) Å] hydrogen bonds.

### Raman spectroscopy

**Figure 3:**
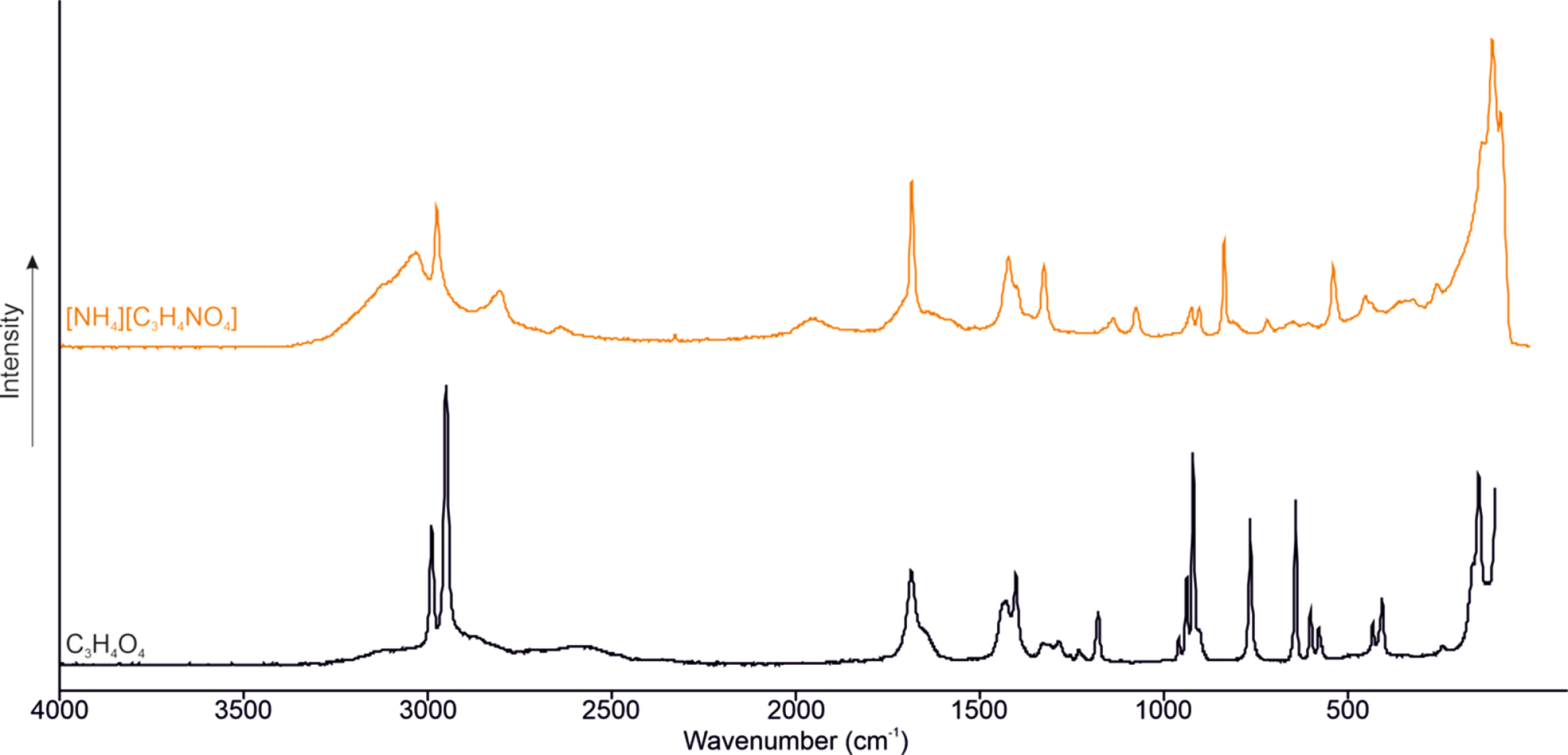
The low-temperature Raman spectrum of malonic acid and (I).

The Raman spectrum of (I) is shown in Fig. 3 ▸, together with that of the starting material malonic acid. The N—H stretching vibrations are detected at 3032 and 2809 cm^−1^. The C—H stretching vibration is observed at 2977 cm^−1^. The polarized C=O stretching vibration is detected at 1684 cm^−1^ and that of C—O at 1328 cm^−1^.

### NMR spectroscopy

**Figure 4:**
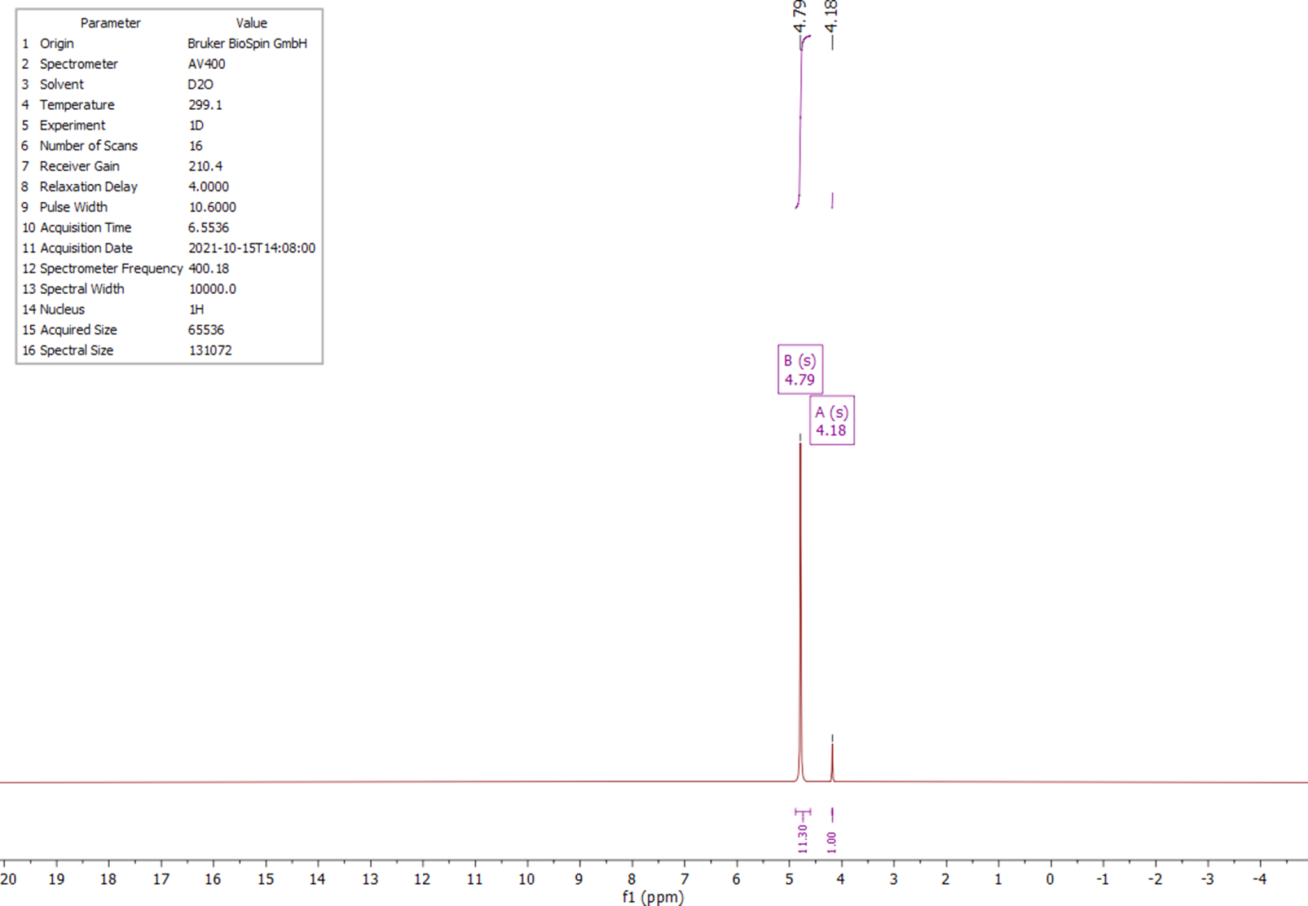
The ^1^H NMR spectrum of (I) in D_2_O.

**Figure 5:**
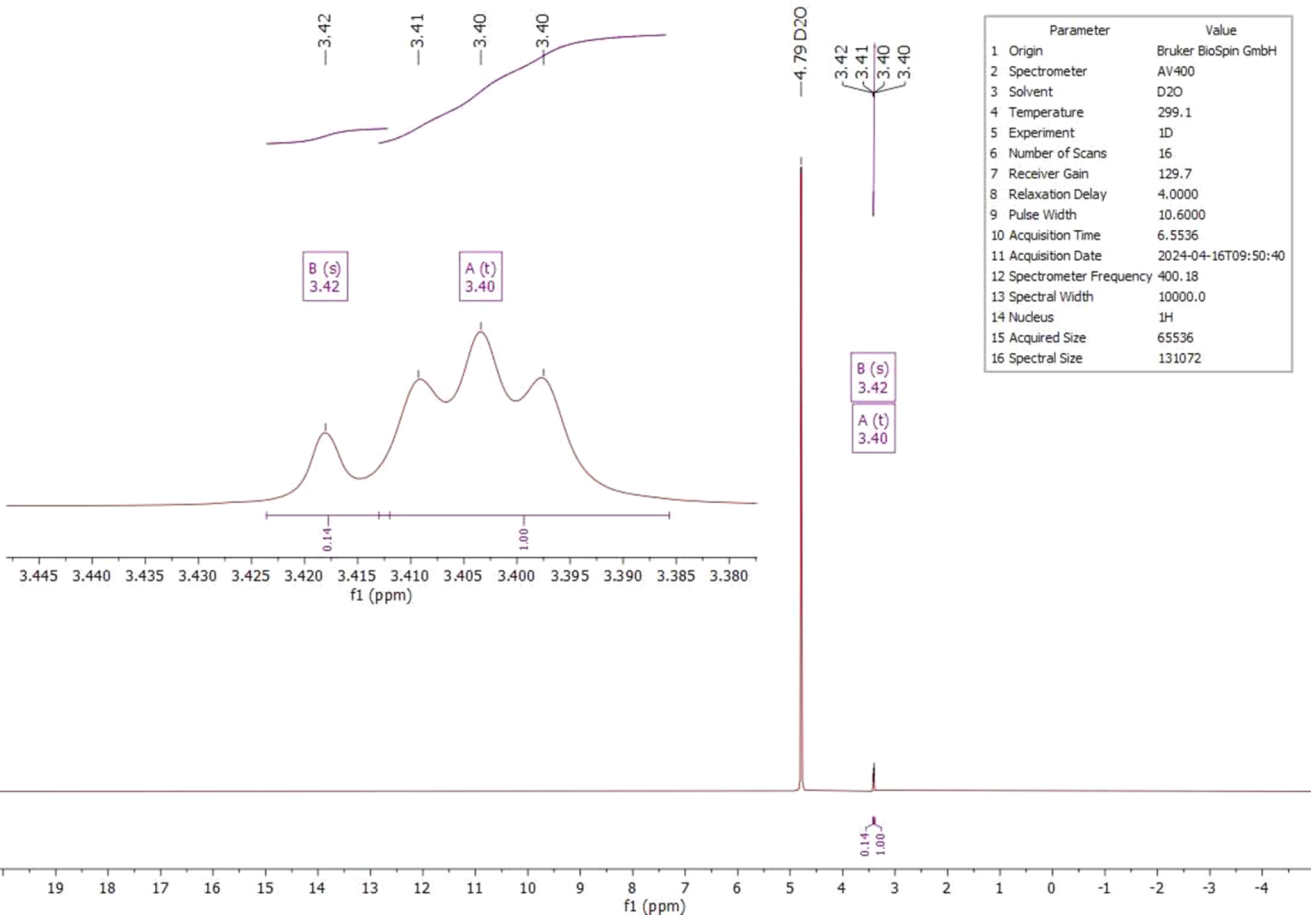
The ^1^H NMR spectrum of malonic acid (C_3_H_4_O_4_) in D_2_O.

**Figure 6:**
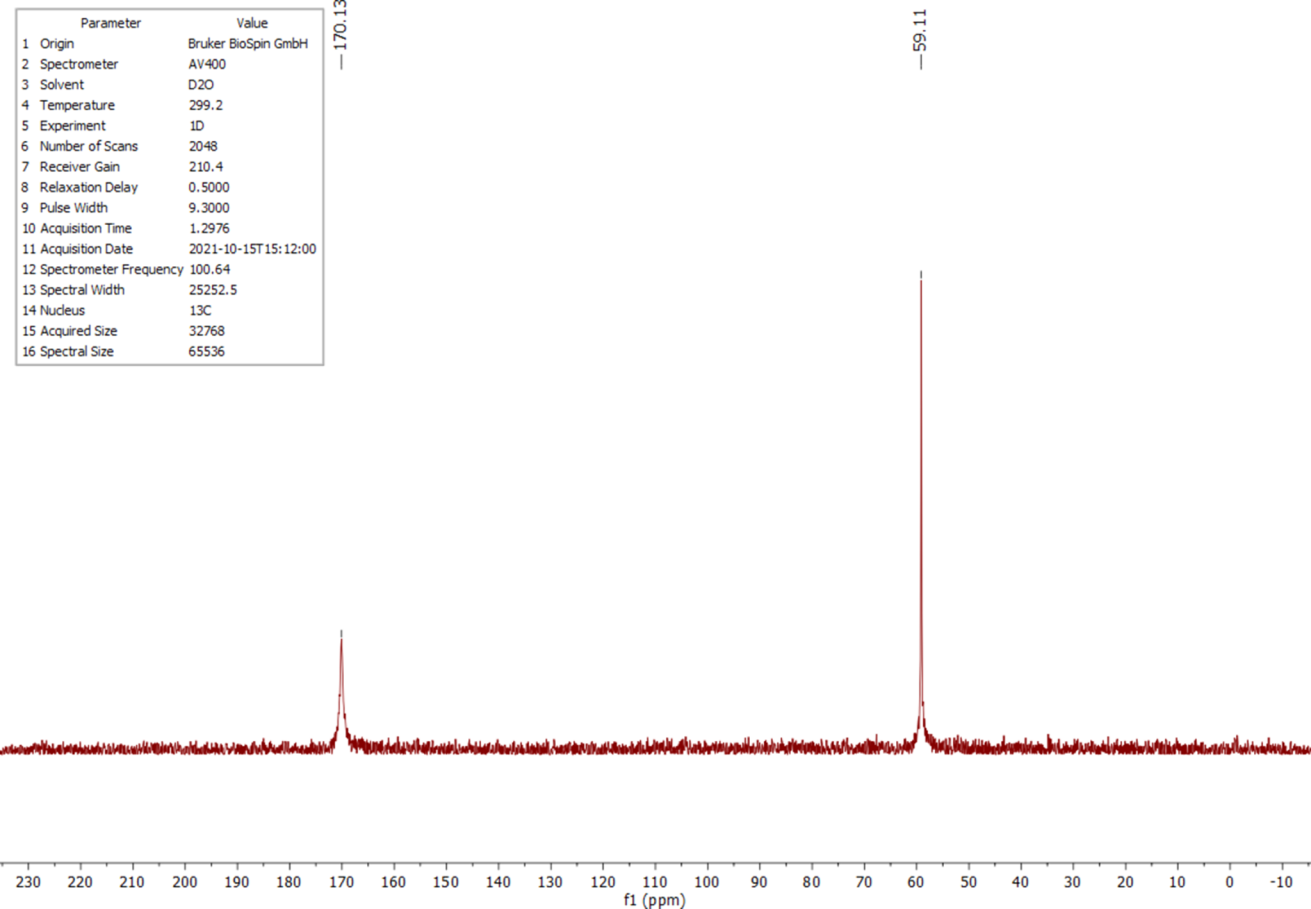
The ^13^C NMR spectrum of (I) in D_2_O.

**Figure 7:**
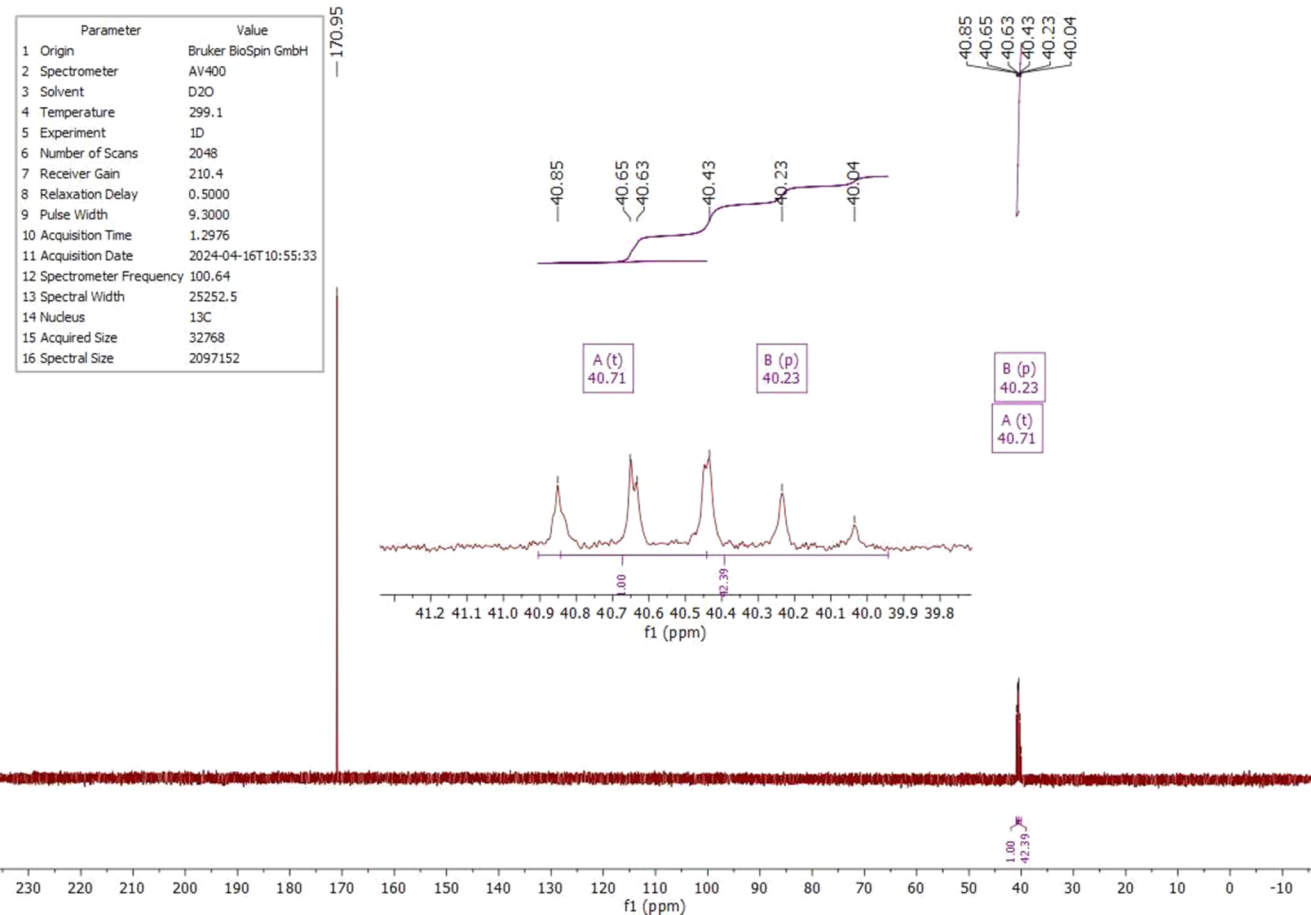
The ^13^C NMR spectrum of malonic acid (C_3_H_4_O_4_) in D_2_O.

**Figure 8:**
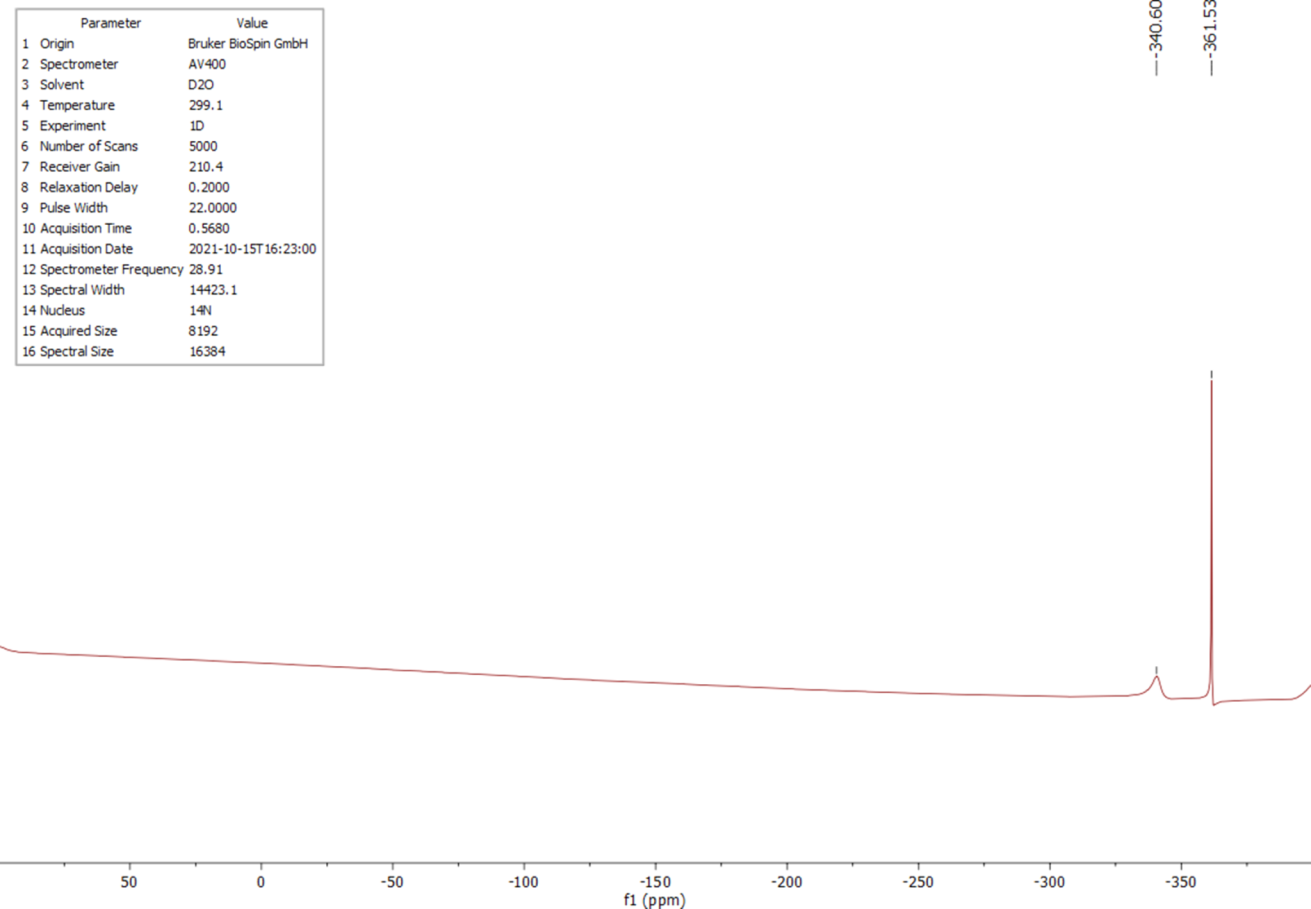
The ^14^N NMR spectrum of (I) in D_2_O.

The ^1^H, ^13^C and ^14^N NMR spectra of salt (I) were measured in D_2_O at room temperature. The ^1^H NMR spectrum (Fig. 4 ▸) shows one singlet at 4.18 ppm (*s*, CH). Compared to the starting material, the proton is significantly less acidic and deshielded by 0.76 ppm. The starting material has an H/D exchange in D_2_O, which is recognizable by the triplet at 3.40 ppm and the singlet at 3.42 ppm (Fig. 5 ▸). The ^13^C NMR analysis of (I) detected the carboxyl C atom at 170.1 ppm and the C2 atom at 59.1 ppm (Fig. 6 ▸); compared to the starting material, the carb­oxy moieties are not significantly shifted (Fig. 7 ▸). The protons of atom C2 of the malonic acid are much more acidic, resulting in the ^13^C NMR spectrum in a triplet at 40.7 ppm (*t*, *J* = 20.0 Hz) and a quintet at 40.2 ppm (*p*, *J* = 20.3 Hz) splitting. In salt (I), the C2 carbon is much more deshielded and a singlet is seen at 59.1 ppm. The ^14^N NMR spectrum (Fig. 8 ▸) shows the ammonium cation at −340.6 ppm and the –NH_3_^+^ moiety at −361.5 ppm as singlets.

## Conclusion

Herein we present the first single-crystal X-ray diffraction and Raman and NMR spectroscopy study of the salt ammonium 2-am­ino­mal­on­ate. For 2-amino­malonic acid, only the ^1^H NMR spectrum is known in the literature (Callahan & Wolfenden, 2004 ▸). Also, we describe the H/D exchange of the CH_2_ moiety in D_2_O of malonic acid for the first time.

## Supplementary Material

Crystal structure: contains datablock(s) I, global. DOI: 10.1107/S2053229624005576/wv3014sup1.cif

Structure factors: contains datablock(s) I. DOI: 10.1107/S2053229624005576/wv3014Isup2.hkl

CCDC reference: 2361889

## Figures and Tables

**Table 1:** Experimental details

Crystal data	
Chemical formula	NH_4_^+^·C_3_H_4_NO_4_^−^
*M*_r_	136.11
Crystal system, space group	Orthorhombic, *Pbca*
Temperature (K)	101
*a*, *b*, *c* (Å)	9.9714 (4), 9.8671 (3), 11.1884 (4)
*V* (Å^3^)	1100.81 (7)
*Z*	8
Radiation type	Mo *K*α
μ (mm^−1^)	0.15
Crystal size (mm)	0.73 × 0.60 × 0.51

Data collection	
Diffractometer	Rigaku Xcalibur Sapphire3
Absorption correction	Multi-scan (*CrysAlis PRO*; Rigaku OD, 2020 ▸)
*T*_min_, *T*_max_	0.847, 1.000
No. of measured, independent and observed [*I* > 2σ(*I*)] reflections	18831, 1483, 1391
*R*_int_	0.021
(sin θ/λ)_max_ (Å^−1^)	0.685

Refinement	
*R*[*F*^2^ > 2σ(*F*^2^)], *wR*(*F*^2^), *S*	0.039, 0.113, 1.20
No. of reflections	1483
No. of parameters	114
H-atom treatment	All H-atom parameters refined
Δρ_max_, Δρ_min_ (e Å^−3^)	0.49, −0.21

Computer programs: *CrysAlis PRO* (Rigaku OD, 2020 ▸), *SHELXT2018* (Sheldrick, 2015*a* ▸), *SHELXL2019* (Sheldrick, 2015*b* ▸), *ORTEP-3* (Farrugia, 2012 ▸) and *PLATON* (Spek, 2020 ▸).
